# Precise Genome Modification via Sequence-Specific Nucleases-Mediated Gene Targeting for Crop Improvement

**DOI:** 10.3389/fpls.2016.01928

**Published:** 2016-12-20

**Authors:** Yongwei Sun, Jingying Li, Lanqin Xia

**Affiliations:** Institute of Crop Sciences (ICS), Chinese Academy of Agricultural Sciences (CAAS)Beijing, China

**Keywords:** clustered regularly interspersed short palindromic repeats (CRISPR)/Cas9, crops, double strand breaks (DSBs), gene targeting (GT), homology-directed repair (HDR), sequence-specific nucleases (SSNs), transcription activator-like effector nucleases (TALENs), zinc finger nucleases (ZFNs)

## Abstract

Genome editing technologies enable precise modifications of DNA sequences *in vivo* and offer a great promise for harnessing plant genes in crop improvement. The precise manipulation of plant genomes relies on the induction of DNA double-strand breaks by sequence-specific nucleases (SSNs) to initiate DNA repair reactions that are based on either non-homologous end joining (NHEJ) or homology-directed repair (HDR). While complete knock-outs and loss-of-function mutations generated by NHEJ are very valuable in defining gene functions, their applications in crop improvement are somewhat limited because many agriculturally important traits are conferred by random point mutations or indels at specific loci in either the genes’ encoding or promoter regions. Therefore, genome modification through SSNs-mediated HDR for gene targeting (GT) that enables either gene replacement or knock-in will provide an unprecedented ability to facilitate plant breeding by allowing introduction of precise point mutations and new gene functions, or integration of foreign genes at specific and desired “safe” harbor in a predefined manner. The emergence of three programmable SSNs, such as zinc finger nucleases, transcriptional activator-like effector nucleases, and the clustered regularly interspaced short palindromic repeat (CRISPR)/CRISPR-associated protein 9 (Cas9) systems has revolutionized genome modification in plants in a more controlled manner. However, while targeted mutagenesis is becoming routine in plants, the potential of GT technology has not been well realized for traits improvement in crops, mainly due to the fact that NHEJ predominates DNA repair process in somatic cells and competes with the HDR pathway, and thus HDR-mediated GT is a relative rare event in plants. Here, we review recent research findings mainly focusing on development and applications of precise GT in plants using three SSNs systems described above, and the potential mechanisms underlying HDR events in plant cells. We then address the challenges and propose future perspectives in order to facilitate the implementation of precise genome modification through SSNs-mediated GT for crop improvement in a global context.

## Introduction

Genome editing has become a powerful tool for functional genomics research in plants and genetic improvement of agricultural crops through precise manipulation of plant genomes. This relies on the creation of targeted DNA double strand breaks (DSBs) by sequence-specific nucleases (SSNs) at specified genomic locations, which will stimulate the cell’s DNA repair machinery. To date, four classes of SSNs, meganucleases/homing endonucleases which in general refer to I-SceI or I-CreI, zinc finger nucleases (ZFNs), transcriptional activator-like effector nucleases (TALENs), and the clustered regularly interspaced short palindromic repeat (CRISPR)/CRISPR-associated protein 9 (Cas9), have been developed to cleave target genes of interest. Upon induction of a DNA DSB, the subsequent repair process in eukaryotic cells predominantly goes through the error-prone non-homologous end joining (NHEJ) pathway to create random indels, leading to frameshift mutations and target gene knockout. The presence of a donor DNA containing sequences homologous to those flanking the DSB site can greatly increase the chance of a precise DSB repair through the homology-directed repair (HDR) pathway, leading to gene replacement or foreign gene cassette knock-in as intended (Puchta, 2005; Puchta and Fauser, 2014; Voytas and Gao, 2014; Baltes and Voytas, 2015). Whereas complete knock-outs and loss-of-function mutations are very valuable in defining gene functions, deciphering complex metabolite pathways and becoming routine in plants (Lawrenson et al., 2015; Ma et al., 2015), their applications in crop improvement are somewhat limited because many agriculturally important traits are conferred by the random point mutations or indels at specific loci in either the genes’ encoding or promoter regions, change of gene expression levels or insertion of a new gene (Kumar et al., 2016; Sun et al., 2016). So far, target gene replacement or gene targeting (GT) has not yet been well established as a feasible technique in higher plants. The primary barrier is the high frequency of illegitimate recombination by which DNA integrates at non-homologous sites (Steinert et al., 2016). NHEJ is the primary pathway involved in DNA repair process in the somatic cells, while HDR mainly occurs during S and G2 phases of the cell cycle (Puchta, 2005). When DNA is introduced into plant cells, the frequency of illegitimate recombination events are typically 10^5^–10^7^ times higher than that of homologous recombination (Paszkowski et al., 1988; Lee et al., 1990; Offringa et al., 1990; Halfter et al., 1992; Hrouda and Paszkowski, 1994; Miao and Lam, 1995; Risseeuw et al., 1995). As a result, the frequency of replacement or targeted integration via HDR is much lower in comparison to random integration (Symington and Gautier, 2011), and reports describing successful gene replacement or site-specific trait gene integration through SSNs-mediated HDR in plants are very limited. Several reviews have described the rapid development and applications of the SSNs system in plants (Voytas and Gao, 2014; Belhaj et al., 2015; Osakabe and Osakabe, 2015; Kumar et al., 2016; Ma et al., 2016; Schiml and Puchta, 2016; Weeks et al., 2016). Here, we present an overview of the recent research advances mainly focusing on development and applications of precise GT in plants using the three recently developed SSN systems, ZFNs, TALENs, and CRISPR/Cas9 reagents, and the potential mechanisms underlying HDR events in plant cells as well as the challenges and future perspectives in implementing precise genome modification through SSNs-mediated GT for crop improvement.

## Ssns-Mediated Gene Targeting in Plants

Ever since HDR was demonstrated to be feasible in plant cells in 1988 (Paszkowski et al., 1988), various classical GT strategies have been attempted to achieve HDR in plants (Paszkowski et al., 1988; Offringa et al., 1990; Zhu et al., 2000; Terada et al., 2002; Okuzaki and Toriyama, 2004; Shaked et al., 2005; Nishizawa-Yokoi et al., 2015). The induction of a DSB at a specific locus can significantly increase the frequency of homologous recombination up to more than 100-fold (Puchta et al., 1996). By using of a site-specific synthetic nuclease, meganuclease I-SceI, to induce DSB at the target locus, HDR events were successfully generated in several plant species, such as *Arabidopsis*, tobacco, rice, and tomato (Puchta et al., 1993, 1996; Beetham et al., 1999; Reiss et al., 2000; Siebert and Puchta, 2002; Fauser et al., 2012; Kwon et al., 2012). The emergence of three programmable SSNs, such as ZFNs, TALENs, and CRISPR/Cas9 has revolutionized the precise genome modification in a more controlled manner in plants. Over the last several years, GT has been achieved in higher plants with a varied degree of success (**Table 1**). Below, we summarized the recent research findings in precise GT in higher plants using the three different SSN systems described above.

**Table 1:** Gene targeting in diverse plant species by employing different sequence-specific nucleases (SSNs).

Method	Plant species	Target gene	Donor	Delivery method	Homology-directed repair (HDR) event	Gene targeting (GT) phenotype	Reference
ZFNs	*Nicotiana tabacum*	Defective *β-Glucuronidase* gene (*GUS):NPTII* (transgene)	Restoring GUS function	Bombardment	Yes	GUS expression	Wright et al., 2005
	*Arabidopsis thaliana*	*PRps5a-gfp/gus cassette* (transgene)	*Hpt* coding region replaced by *gfp* coding region	*Agrobacterium*	Yes	GFP expression	de Pater et al., 2009
	*Nicotiana tabacum*	Endo-chitinase gene *CHN50*	*PAT* expression cassette	*Agrobacterium*	Yes	Herbicide resistances	Cai et al., 2009
	*Nicotiana tabacum*	*ALS*	*ALS* with mutant site	Electroporation	Yes	Herbicide resistances	Townsend et al., 2009
	*Zea mays*	*IPK1*	*PAT* expression cassette/*PAT* gene no promoter	Silicon carbide whiskers	Yes	Herbicide resistances	Shukla et al., 2009
	*Arabidopsis thaliana*	Mutated *GUS* expression cassette (transgene)	No donor	*Agrobacterium*	Yes	GUS expression	Even-Faitelson et al., 2011
	*Arabidopsis thaliana*	Protoporphyrinogen oxidase (PPO)	T-DNA harboring an incomplete *PPO* gene	*Agrobacterium*	Yes	Insensitive to the herbicide butafenacil	de Pater et al., 2013
	*Arabidopsis thaliana*	*ADH1*	*ADH1* fragment with a insertion of 68 bp and deletion of 12 bp	*Agrobacterium*	Yes	N.A	Qi et al., 2013
	*Arabidopsis thaliana*	Quasipalindromic QQR ZFN recognition sites	Promoter-less *hpt* gene	*Agrobacterium*	Yes	Hygromycin resistance	Weinthal et al., 2013
	*Nicotiana tabacum*	Quasipalindromic QQR ZFN recognition sites	Promoter-less *hpt* gene	*Agrobacterium*	Yes	Hygromycin resistance	Weinthal et al., 2013
	*Zea mays*	*TLPs*	AAD1 expression cassette	Bombardment	Yes	Herbicide resistances	Ainley et al., 2013
	*Nicotiana tabacum*	Defective *GUS* (transgene)	Restoring gus:nptII gene (Geminivirus-based replicons)	*Agrobacterium*	Yes	GUS expression and kanamycin resistance	Baltes et al., 2014
TALENs	*Nicotiana tabacum*	*ALS*	ALS with mutant site	PEG	No	N.A	Zhang et al., 2013
	*Hordeum vulgare*	*GFP* (transgene)	Truncated *yfp* fragment	Bombardment	No	YFP expression	Budhagatapalli et al., 2015
	*Solanum lycopersicum*	Anthocyanin mutant 1 (*ANT1*)	35S promoter upstream of the endogenous *ANT1* coding sequence	*Agrobacterium*	Yes	Purple plant tissue	Cermak et al., 2015
	*Solanum tuberosum*	*ALS1*	ALS with mutant site (Geminivirus-based replicons)	*Agrobacterium*	Yes	Herbicide resistances	Butler et al., 2016
	*Oryza sativa*	*ALS*	*ALS* with mutant site	Bombardment	Yes	Herbicide resistances	Li et al., 2016
CRISPR/Cas9	*Arabidopsis thaliana*	*DGU.US, IU.GUS* (transgene)	No donor	*Agrobacterium*	Yes	GUS expression	Fauser et al., 2014
	*Arabidopsis thaliana*	*ADH1*	*NptII* expression cassette	*Agrobacterium*	Yes	Kanamycin resistance	Schiml et al., 2014
	*Solanum lycopersicum*	Anthocyanin mutant 1 *(ANT1)*	35S promoter upstream of the endogenous *ANT1* coding sequence (Geminivirus-based replicons)	*Agrobacterium*	Yes	Purple plant tissue	Cermak et al., 2015
	*Glycine max*	*ALS1*	*ALS* with mutant site	Bombardment	No	N.A	Li et al., 2015
	*Glycine max*	Genomic sites DD43 on chromosome 4	*Hpt* expression cassette	Bombardment	Yes	Hygromycin resistances	Li et al., 2015
	*Zea mays*	*ALS2*	*ALS* with mutant site	Bombardment	Yes	Herbicide resistances	Svitashev et al., 2015
	*Zea mays*	Liguleless-1 (LIG1)	*PAT* expression cassette	Bombardment	Yes	Herbicide resistances	Svitashev et al., 2015
	*Linum usitatissimum*	*EPSPS*	*EPSPS* with mutant site	PEG	Yes	Herbicide resistances	Sauer et al., 2016
	*Solanum tuberosum*	*ALS1*	*ALS* with mutant site (Geminivirus-based replicons)	*Agrobacterium*	Yes	Herbicide resistances	Butler et al., 2016
	*Zea mays*	*ARGOS8*	*GOS2* promoter	Bombardment	Yes	Improved grain yield under drought stress conditions	Shi et al., 2016
	*Oryza sativa*	*ALS*	*ALS* with mutant site	*Agrobacterium*	Yes	Herbicide resistances	Endo et al., 2016
	*Oryza sativa*	*ALS*	*ALS* with mutant site	Bombardment	Yes	Herbicide resistances	Sun et al., 2016

*HDR event, obtained plants through HDR.*

### GT in Plants Using ZFNs

**FIGURE 1:**
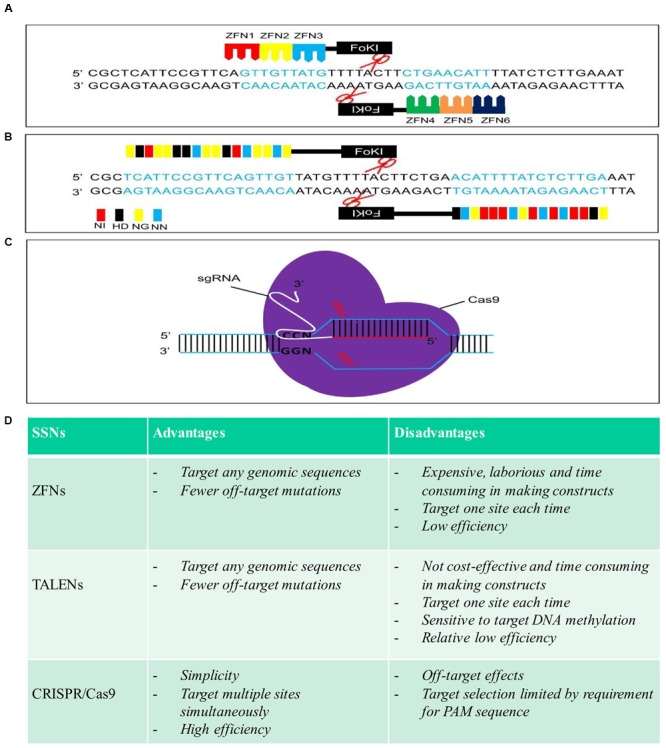
**Schematic structures, advantages, and disadvantages of zinc finger nucleases (ZFNs), transcription activator-like effector nucleases (TALENs), and clustered regularly interspaced short palindromic repeat (CRISPR)/Cas9. (A), (B)**, and **(C)** are schematic structures of ZFNs, TALENs, and CRISPR/Cas9 in the process of DNA cleavage, respectively. **(D)** Advantages and disadvantages of each sequence-specific nuclease (SSN).

Zinc finger nucleases, as the first generation of SSNs, were used to edit plant genomes (Smith et al., 2000; Bibikova et al., 2002; Lloyd et al., 2005; Zhang et al., 2010; Zhang and Voytas, 2011; Kumar et al., 2015; Petolino, 2015). By fusing the DNA cleavage domain from the restriction enzyme *Fok*I to the highly variable DNA binding domain (DBD) of different zinc finger transcription factors to form different ZFNs, different target sites in the DNA can be recognized and cleaved (**Figure 1A**) (Kim et al., 1996). GT was demonstrated to be feasible in *Arabidopsis* and tobacco by inducing a DSB with a ZFN (Wright et al., 2005; Cai et al., 2009; de Pater et al., 2009, 2013; Townsend et al., 2009; Even-Faitelson et al., 2011; Qi et al., 2013; Weinthal et al., 2013; Baltes et al., 2014). So far, only two cases reported the successful GT for integration or stacking herbicide resistances gene(s) in a crop plant (maize). For example, simultaneous expression of ZFNs and delivery of a heterologous donor molecule led to precise targeted insertion of an herbicide tolerance gene expression cassette at the inositol 1,3,4,5,6-pentakisphosphate 2-kinase (IPK1) locus in maize (Shukla et al., 2009). Combination of the engineered ZFNs with modular “trait landing pads” (TLPs) could enable the site-specific transgene integration and traits stacking in crop plants (Ainley et al., 2013). For example, an herbicide resistance gene, phosphinothricin acetyltransferase (*pat*), along with TLPs was integrated into maize genome in the first round of transformation. Then, a second herbicide resistance gene, aryloxyalkanoate dioxygenase (*aad1*), flanked by sequences homologous to the integrated TLPs, along with a corresponding ZFN expression construct, was precisely targeted to the genomic locus of previous integrated *pat* following a second round of transformation, resulting in a sequential stack. Up to 5% of the embryo-derived transgenic events contained the *aad1* transgene integrated precisely at the TLP which was directly adjacent to the *pat* transgene (Ainley et al., 2013). The ability to stack multiple trait genes at a single locus by ZFNs-mediated GT to enable simple inheritance addresses a significant agricultural challenge.

### GT in Plants Using TALENs

Over the past few years, TALENs have emerged as the reagent of choice in genome engineering in plants (Bogdanove and Voytas, 2011). Like ZFNs, TALENs are chimeric proteins produced by fusing an engineered DNA-binding domain with the catalytic domain of FokI endonuclease, which cleaves as a dimer (**Figure 1B**) (Christian et al., 2010; Li et al., 2011). TALENs and ZFNs, therefore, work in a same way in that two monomers bind opposing strands of DNA separated by a spacer of an appropriate length, allowing FokI to dimerize and cleave DNA. One of the advantages of TALENs over ZFNs is that the DBD can be easily engineered to recognize virtually any DNA sequence (Miller et al., 2011). So far, TALENs have been applied successfully for genome editing of a variety of different plant species, such as *Arabidopsis*, brachypodium, tobacco, tomato, rice, barley, wheat, and maize (Mahfouz et al., 2011; Shan et al., 2013; Zhang et al., 2013; Wang et al., 2014; Budhagatapalli et al., 2015; Cermak et al., 2015; Butler et al., 2016; Li et al., 2016), among which, only a few cases reported the recovery of precisely edited plants in GT experiments (**Table 1**). For example, TALENs introduced targeted mutations in acetolactate synthase (ALS) in 30% of transformed cells in tobacco protoplast, and the frequencies of targeted gene insertion reached at 14% (Zhang et al., 2013). The feasibility of precise modification of a target DNA sequence which resulted in a predicted alteration of gene function was also demonstrated in barley, when green fluorescent protein gene (*gfp*) specific TALENs along with a repair template were introduced into barley calli, conversion of *gfp* into yellow fluorescent protein gene (*yfp*) via HDR was achieved in three of 100 calli bombarded (Budhagatapalli et al., 2015). A strong promoter was inserted upstream of a gene controlling anthocyanin biosynthesis, precise modification of the tomato genome was achieved through either TALENS or CRISPR/Cas9 using geminivirus replicons, resulting in overexpression and ectopic accumulation of pigments in tomato tissues (Cermak et al., 2015). The same strategy was also successful applied in generation of herbicide resistant potato plants by introducing one point mutation in potato *ALS1* gene through gene replacement (Butler et al., 2016). Recently, through *Agrobacterium*-mediated transformation, herbicide resistant rice plants were recovered by introducing double point mutations in rice *ALS* through TALENs-mediated gene replacement with an efficiency of 1.4–6.3% (Li et al., 2016).

However, it is worth to mention that both ZFNs and TALENs need tandem repeats in their DNA-binding domains that engineered to recognize specific DNA sequences in the genome to generate DSBs. In this case, a new chimeric protein must be engineered for each new target sequence of interest (**Figures 1A,B**). This has been a major hurdle to the wider application of these two SSNs because engineering new protein is a very complicate process, non-cost effective, time-consuming and is not feasible in most laboratories (**Figure 1D**).

### GT in Plants Using CRISPR/Cas9

As a third generation of designed SSNs, the emergence of CRISPR/Cas9 has revolutionized genome editing because of its specificity, simplicity, and versatility (Cong et al., 2013; Feng et al., 2014; Gao and Zhao, 2014; Zhou et al., 2014; Lawrenson et al., 2015; Ma et al., 2015, 2016; Xie et al., 2015; Sun et al., 2016). The CRISPR/Cas9 system uses a single guide RNA (sgRNA) to direct the Cas9 endonuclease to the complementary target DNA, and only a new sgRNA is needed for a new target site of interest whilst the nuclease itself remains unmodified (**Figure 1C**) (Jinek et al., 2012; Gaj et al., 2013). Thus, the CRISPR/Cas9 system surpasses ZFNs and TALENs, for its simplicity, versatility and high efficiency, and has been successfully applied in precise genome modification in many organisms including plants (**Figure 1D**) (Doudna and Charpentier, 2014; Ma et al., 2016; Weeks et al., 2016). However, the majority of these studies in plants reported genome editing via NHEJ to generate random loss-of-function mutations or gene knock-outs (Ma et al., 2016). Although gene replacement or GT could be potentially achieved through HDR after CRSIPR/Cas9 generates a DSB at specific gene loci, it remains very challenging to make use of HDR in plants through CRISPR/Cas9-mediated genome editing (Svitashev et al., 2015; Sun et al., 2016). The successful gene replacement or GT has been documented so far in a limited number of studies in *Arabidopsis*, rice, maize and flax (*Linum usitatissimum*) (**Table 1**). The CRISPR/Cas9 system can be used as nuclease for in planta GT in *Arabidopsis* (Schiml et al., 2014). ALS is a key enzyme for the biosynthesis of branched chain amino acids and which is a major target for agriculturally important herbicides including chlorsulfuron and bispyribac-sodium (BS). Substitution of proline 165 with serine in the *ALS2* gene using either single-stranded oligonucleotides or double-stranded DNA vectors as repair templates yielded chlorsulfuron resistant maize plants via CRISPR/Cas9-mediated gene editing. However, the efficiency of generation of herbicide resistant plants was very low. Among 1000 calli bombarded, only nine calli recovered were able to regenerate herbicide resistant plants (Svitashev et al., 2015). In another parallel experiment with HDR-mediated gene insertion at an endogenous *liguleless-1* gene (L1G) target site in maize, co-delivery of both Cas9-sgRNA and donor DNA either separately or as a single vector through bombardment resulted in a frequency of 2.5–4% of target insertion, respectively (Svitashev et al., 2015). A repair construct and the CRISPR/Cas9 expression vector targeting the rice *ALS* gene were transferred into rice calli either separately or sequentially through *Agrobacterium*-mediated transformation, resulted in herbicide resistant rice plants with the ratio of GT frequency of 0.323% (Endo et al., 2016). In our previous study, by using dual sgRNAs in combination with two sources of DNA repair templates, one released from T-DNA in planta and the other co-bombarded as free double-stranded DNA, to promote HDR-mediated *ALS* gene replacement in rice, multiple homozygous herbicide resistant rice plants in T_0_ generation were successfully recovered via CRISPR/Cas9-mediated HDR for simultaneous in planta point substitutions of two amino acid residues, tryptophan 548 and serine 627 in the rice ALS with leucine and isoleucine (Sun et al., 2016). Maize *ARGOS8* is a negative regulator of ethylene responses. The maize *GOS2* promoter was inserted into the 5’ untranslated region of the native *ARGOS8* gene to replace the native promoter of *ARGOS*8 through CRISPR/Cas9-mediated HDR. Precise promotor replacement at the *ARGOS8* locus resulted in increased grain yield by five bushels per acre under stress conditions (Shi et al., 2016). An herbicide tolerance trait was also developed in flax (*L. usitatissimum*) by precise modification of the 5-enolpyruvylshikimate-3-phosphate synthase (EPSPS) gene through a co-delivery of CRISPR/Cas9 system and the single-stranded oligonucleotides (ssODN) template into protoplast with the frequency of precise EPSPS edited events ranged between 0.09 and 0.23% (Sauer et al., 2016). Most recently, through bombardment of a vector harboring the CRISPR/Cas9 system and donor template, glyphosate-resistant rice plants was generated by CRISPR/Cas9-mediated gene replacement of the intron region of *EPSPS* gene at an efficiency of 2.0% (Li et al., 2016).

## The Potential Mechanisms Underlying HDR Inside Plant Cells

**FIGURE 2:**
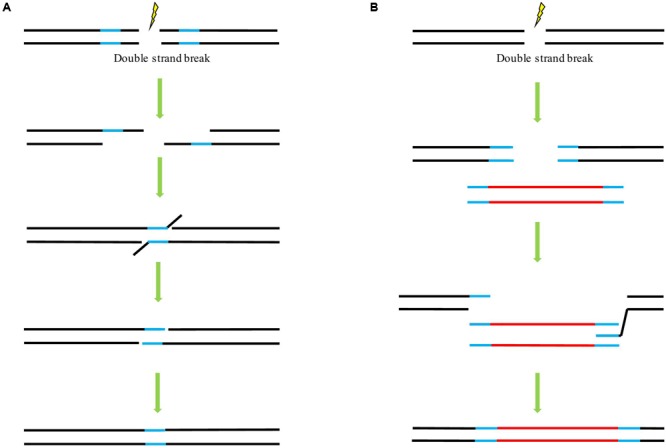
**The potential mechanisms underlying double strand break (DSB) repair upon the availability of a homologous template. (A)** Single-strand annealing (SSA) pathway of homology-directed repair (HDR). **(B)** Synthesis-dependent strand annealing (SDSA) pathway of HDR. Lines in blue indicate the homologous sequences. Lines in red indicate the foreign sequences.

In principle, there are three main mechanisms of DSB repair involving the use of a homologous template: single-strand annealing (SSA) (**Figure 2A**), synthesis-dependent strand annealing (SDSA) (**Figure 2B**), and the so-called double-strand break repair (DSBR) model (Puchta and Fauser, 2014). Following DSB induction in all pathways, single-stranded overhangs are produced via exonuclease-catalyzed resection. In the case of the SSA mechanism, both ends of the break carry complementary sequences. These molecules can then anneal to one another to form a chimeric DNA molecule with the 3’-overhangs be trimmed. As a consequence, the sequences flanking the complementary sequences will be lost (Puchta and Fauser, 2014). SSA can, in principle, also occur between two DNA molecules that are not linked. These molecules could be transfected plasmid DNAs or T-DNAs as well as broken chromosomes (Puchta and Hohn, 1991a; Tinland et al., 1994; Pacher et al., 2007). This mechanism is not conservative since all sequence information between the respective repeats is lost, SSA can proceed in somatic plant cells as efficient as NHEJ (Knoll et al., 2012). In the case of DSBR and SDSA, the homologous repair template can be supplied in *cis* or *trans*. Following the DSB induction, 3’-end invasion of a single strand into a homologous double strand occurs, resulting in a D-loop (**Figure 2B**). Reparative synthesis is initiated using the newly paired strand as a template. Whereas in SDSA, the genetic information of a homologous sequence is only copied to one strand, leading to no loss of sequence information although the reaction sometimes results in gene conversion (Puchta and Fauser, 2013), in DSBR, DNA synthesis occurs at both broken ends, respectively, so that genetic information is copied from both strands of the homologous sequences, respectively, thus may lead to a crossover event. DSBR is a prominent mechanism for meiotic recombination (Osman et al., 2011). In our previous study with *ALS* gene replacement in rice, evaluation of the HDR events demonstrated that while most of the HDR events faithfully copied the genetic information of the donor template, some only carried the substitutions at 5′-end and some harbored the conversion at the 3′-end, and no crossover detected in our case (Sun et al., 2016). Our result is in consistence with other GT experiments in which SDSA may represent the major class of GT events and is probably a predominant mechanism underlying HDR event as well as to a combination of HDR and NHEJ events (Puchta et al., 1996; Reiss et al., 2000; Wright et al., 2005). As the SDSA pathway is beneficial for genome stability, it seems to be a predominant pathway responsible for conservative HDR in somatic plant cells (Puchta, 1998; Osman et al., 2011). So far, a majority of successful GT experiments employed the DNA repair template flanked with homologous arms at each end. A positive correlation was found between the HDR rates and the lengths of overlapping homology (up to 1200 bp) of the transfected supercoiled circular or linearized plasmids, with a significantly decreased HDR rate was observed when the overlap of both substrates was reduced to 456 bp or less (Puchta and Hohn, 1991b).

## Challenges and Future Perspectives

**FIGURE 3:**
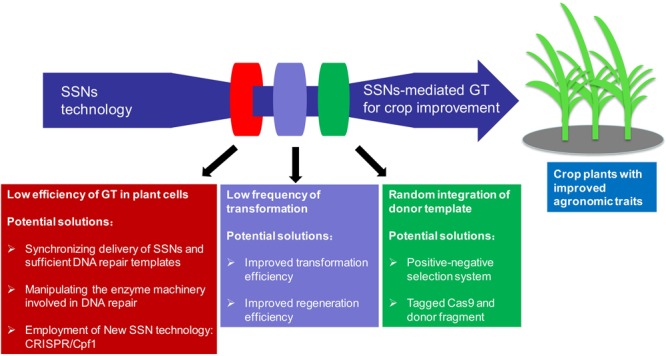
**Current challenges and future perspectives in applying SSNs-mediated gene targeting (GT) for crop improvement**.

One major obstacle in performing GT for crop improvement is its low efficiency because repair through NHEJ predominates in plant somatic cells and competes with the HDR pathway (**Figure 3**) (Puchta et al., 1996; Qi et al., 2013). Other challenges including plant species recalcitrance to tissue culture and transformation, and thus low frequency of stably transformed events limit efforts to use genome editing for genetic improvement in some crop species and genotypes (**Figure 3**) (Shrawat and Lorz, 2006; Hiei et al., 2014; for review, please see Altpeter et al., 2016). Moreover, unintended random integration of donor template should be identified, suppressed or segregated because of biosafety concerns in application of these edited crop plants in a breeding practice (**Figure 3**) (Altpeter et al., 2016; Kumar et al., 2016). Consequently, substantial efforts have been devoted in the last few years to meet the above challenges.

### Synchronizing Delivery of SSNs and Sufficient DNA Repair Templates

In theory, synchronizing the DSB induction by SSNs and delivery of the HDR template is essential for the occurrence of HDR in plant cells (Baltes et al., 2014; Schiml et al., 2014; Endo et al., 2016; Sun et al., 2016). To increase the frequency of HDR-mediated GT, timing the induction DSBs on the target gene to coincide with the delivery of sufficient HDR template is crucial for the successful GT events. Therefore, how to deliver the SSNs and DNA repair templates for HDR represent hurdles to the efficient achievement of GT (**Figure 3**). Protoplasts can be transformed with both SSNs and DNA repair template at high efficiency (Wright et al., 2005; Townsend et al., 2009; Zhang et al., 2013), however, for most plant species, especially major cereal crops, regeneration of plants from cultured protoplasts is still not feasible. An efficient way to supply the pant cell with a matrix for HDR-mediated DSB repair is to use an incoming T-DNA from *Agrobacterium tumefaciens* or transfected plasmid DNA (Puchta and Fauser, 2015). A sophisticated system, in planta GT, to enhance gene replacement was first established in 2012 using the meganuclease I-SceI (Fauser et al., 2012), then successfully applied in *Arabidopsis* using the CRISPR/Cas9 reagent by the same group in 2014 (Schiml et al., 2014). It is based on a transgene carrying both sequences homologous to the flanking sequences of the target locus and two recognition sites for a SSN, which also cuts the locus of interest in plant genome. In planta GT system allows the simultaneous release of a linear GT vector and the induction of a DSB at the target locus. Under this strategy, the GT vector can be designed for the site-specific integration of transgenes or to modify the target gene/locus in a predefined manner (Puchta and Fauser, 2015). Considering the low DNA titers delivered by *Agrobacterium*-mediated gene transfer, biolistic gene transfer may be superior for HDR-mediated GT by simultaneously delivery of both SSNs and DNA repair template and providing larger quantities of repair template. By using the same strategy, dual sgRNAs in combination of two sources of DNA repair templates, one released from T-DNA in planta and the other co-bombarded as free double-stranded DNA, to promote HDR-mediated *ALS* gene replacement, multiple homozygous GT rice plants were successfully recovered in T_0_ generation with a highest frequency up to 10%, whereas only hemizygous lines were recovered after two rounds of BS selection in our *Agrobacterium* experiments (Sun et al., 2016). Thus, it would be interesting to investigate the effects of different delivery methods and parameters on GT in crop plants. Furthermore, to overcome the challenges of inefficient transformation and plant regeneration systems in a majority of crop species or genotypes, intra-genomic homologous recombination through intra-genomic mobilization by crossing the SSNs transgenic lines with the transgenic plants harboring stable integrated donor DNA template, is an alternative effective strategy not only for GT, but also for gene stacking (Kumar et al., 2016). In addition, taking advantage of geminivirus replicon, a GT enhancement of greater than two orders of magnitude has been achieved (Baltes et al., 2014). A T-DNA construct harboring the minimal parts necessary for geminivirus replication, a ZFN and a donor template was used to transform tobacco. After transformation, the rolling circle replication of the replicon was initiated at the large intergenic regions (LIRs) that flank the T-DNA construct, leading to the circularization of the construct. Thereafter, the ZFN was expressed and induced a DSB in a defective target gene. GT events occurred using the supplied correct donor template sequence, copying it via GT in the target gene and leading to gene restoration, and thus the donor template was replicated multiple times (Baltes et al., 2014). Heritable gene replacement was achieved by using this strategy in tomato at frequencies 10-fold higher than traditional methods of DNA delivery (i.e., *Agrobacterium*). Both TALENs and CRISPR/Cas9 achieved GT with more than two-thirds of the insertions were precise, and had no unanticipated sequence modifications (Cermak et al., 2015). This method overcomes the efficiency barrier that has made GT in plants challenging.

### Manipulating the Enzyme Machinery Involved in DNA Repair to Enhance GT in Plants

Once DSB induced, DNA repair through NHEJ predominates in somatic cells and competes with the HDR pathway. Suppression of core components of the NHEJ pathway or enhancement of key elements of HDR machinery can be used to increase frequencies of HDR (**Figure 3**) (Puchta et al., 1996; Qi et al., 2013). Expression of a bacterial *RecA* gene in plants stimulated HDR in tobacco (Reiss et al., 2000). During HDR-mediated DSB repair in eukaryotes, creation of a single-stranded DNA (ssDNA) overhang via resection of a 5′ end is an initial step. RAD51 can polymerize on this ssDNA to search for a homologous sequence so that the gapped sequence is then repaired using another undamaged homologous DNA strand as template (Kwon et al., 2012). Therefore, the presence of RAD51 is extremely important for SDSA in *Arabidopsis*, but not for SSA. The same is true for the SWI2/SNF2 chromatin remodeler AtRAD54 (Roth et al., 2012). Expression of a yeast *RAD54* gene led to an increase in GT efficiency in *Arabidopsis* (Shaked et al., 2005; Even-Faitelson et al., 2011). The fact that expression of a rice *OsRecQl4* (a gene encoding bloom helicase counterpart) and/or rice *exonuclease 1* could enhance intra-chromosomal HDR was taken as an indication that these proteins might, in fact, be involved in end resection in plants (Kwon et al., 2012). Indeed, *Arabidopsis* plants with a deficit of *RECQ4A* showed some deficiency in both the SSA and SDSA pathways (Knoll and Puchta, 2011). Instead of heterologous expression of HDR-related proteins, GT efficiency could also be increased by suppression of proteins involved in NHEJ, which led to a hyper-recombination phenotype in *Arabidopsis* (Endo et al., 2006; Hartung et al., 2007; Knoll and Puchta, 2011; Kwon et al., 2012; Recker et al., 2014). DNA repair proteins, such as KU70/80 and Ligase 4 (Lig4) are involved in classic NHEJ, suppression of KU70 or Lig4 function increased the frequency of ZFNs-mediated GT 5- to 16-fold and threefold to fourfold in *Arabidopsis ku70* and *lig4* mutants, respectively (Qi et al., 2013). HDR activity in calli with a *Lig4* deficiency background was twofold to threefold higher than that in control calli in rice. Combination of the induced DSBs via SSNs at a target gene and suppression of NHEJ-related genes or treatment with Lig4 inhibitors can be expected to enhance synergistically the frequency of GT in rice (Endo et al., 2016). However, one obstacle with the manipulation of the HDR repair machinery is that this may lead to a destabilization of the genome, as higher HDR efficiencies can also lead to undesirable recombination events between repetitive sequence motives that are abundantly present in plant species with larger and complex genomes (Steinert et al., 2016).

### Enriching GT Events Using Positive-Negative Selection System

Positive-negative selection (PNS) is an alternative approach to enrich HDR-mediated GT events; it can eliminate NHEJ effectively by expression of a negative-selection marker gene (**Figure 3**) (Shimatani et al., 2015). The single copy *Waxy* locus was targeted for classical GT (knock-in) using a PNS vector carrying the *hygromycin phosphotransferase* gene (*hpt*) for positive selection followed by the effective transcriptional stop signal of the maize transposon En/Spm, which was positioned between the *Waxy* homologous sequences, and one negative selection gene DT-A (diphtheria toxin A-fragment from *Corynebacterium diphtheriae*) flanked with the homologous sequence at both ends (Terada et al., 2002). Since then, the endogenous rice genes at more than 10 loci have been targeted (Terada et al., 2007; Yamauchi et al., 2009; Moritoh et al., 2012; Ono et al., 2012; Ozawa et al., 2012; Dang et al., 2013; Osakabe et al., 2014). Using a combination of neomycin phosphotransferase II (*nptII*) and an antisense *nptII* construct, a universally applicable PNS system for GT in plants was established, although negative selection with this system is relatively less efficient compared with DT-A (Nishizawa-Yokoi et al., 2015). It is worth to note that although this strategy has been only documented in classical GT experiment, it is expected that combination of SSNs-mediated GT with PNS strategy may facilitate the enrichment and recovery of GT events in plants.

### Toward Successful Implementation of Precise Genome Modification through SSNs-Mediated GT for Crop Improvement

Over the last several years, genome manipulation has been revolutionized by the development of three types of SSNs for the control induction of DSBs and thus offers a great promise for harnessing plant genes in crop improvement. However, only a handful of studies reported the precise modification of an endogenous gene for knock-in or target gene replacement in crop plants due to the fact that GT has not been established as a feasible technique in a majority of laboratories. To establish a routine GT system in crop plants, synchronization of DSB induction and delivery of sufficient DNA repair template is crucial for successful GT (Schiml et al., 2014; Endo et al., 2016; Sun et al., 2016). Virus replicon-based GT could also be a good choice to provide sufficient donor template (Baltes et al., 2014; Cermak et al., 2015). For crop species recalcitrant to transformation and regeneration, intra-chromosome HDR will be an effective alternative strategy (Kumar et al., 2016). Furthermore, suppression of core components of the NHEJ pathway or enhancement of key elements of HDR machinery can be used to increase frequencies of GT (Puchta et al., 1996; Qi et al., 2013; Puchta and Fauser, 2015). In addition, to date, most of the GT events reported in plants were either herbicide resistant genes or selectable marker genes in which positive selection for GT is much easier such that precise insertion or gene replacement of the donor in the target locus conferred resistance to selection and avoidance of random integration of the donor (**Table 1**). The PNS selection system might be an attractive strategy to increase the GT efficiency of non-selectable target genes through enrichment of the DSB-induced GT events. Moreover, most recently, a new RNA-guided genome engineering tool, the CRISPR/Cpf1 system, was reported to have properties different from those of the CRISPR/Cas9 systems in that the CRISPR/Cpf1 system has a single RNA-guided endonuclease lacking tracrRNA, 5′ T-rich protospacer adjacent motif (PAM), and a staggered DSB with 4 or 5-nt overhang in contrast to the blunt ends generated by Cas9 (Zetsche et al., 2015). This structure of the cleavage product could be particularly advantageous for facilitating NHEJ-based gene insertion into the plant genome because the DNA insert could be designed to integrate into the genome in a proper orientation (Zetsche et al., 2015). Specifically, Cpf1 could provide an effective way to precisely introduce DNA into the genome via NHEJ mechanism in somatic cells in which genome editing via HDR mechanisms is especially challenging (Chan et al., 2011). At last, regeneration and transformation efficiency, and issues of regulation must also be taken into consideration when selecting a transformation strategy for a given crop species (Altpeter et al., 2016). Both the CRISPR/Cas9 array and the donor templates could be tagged by fluorescence proteins and tracked and eliminated following segregation in the progeny to avoid the random integration of donor fragments and obtain Cas9-free lines (Gao et al., 2016). However, this tagging strategy will only feasible for the integrations of intact constructs. For the integration of fragmented SSN expression cassettes or GT donor molecules, genome-wide sequencing would solve this problem and should not be a major cost issue for sequenced organisms; especially if these developed new varieties will be commercialized in the future. Nonetheless, combination of the different strategies discussed above will be expected to make GT more efficient in crop plants (**Figure 3**). And it is tempting to propose that in the long run, precise genome modification through SSNs-mediated GT will greatly facilitate crop improvement by fully exploiting the agronomic traits important alleles within a gene pool or even beyond species boundaries.

## Author Contributions

YWS and JYL wrote the manuscript; LQX revised the manuscript.

## Conflict of Interest Statement

The authors declare that the research was conducted in the absence of any commercial or financial relationships that could be construed as a potential conflict of interest.

## References

[B1] Ainley W. M. Sastry-Dent L. Welter M. E. Murray M. G. Zeitler B. Amora R. (2013). Trait stacking via targeted genome editing. *Plant Biotechnol. J.* 11 1126–1134. 10.1111/pbi.12107 23953646

[B2] Altpeter F. Springer N. M. Bartley L. E. Blechl A. E. Brutnell T. P. Citovsky V. (2016). Advancing crop transformation in the era of genome editing. *Plant Cell* 28 1510–1520. 10.1105/tpc.16.00196 27335450 PMC4981132

[B3] Baltes N. J. Gil-Humanes J. Cermak T. Atkins P. A. Voytas D. F. (2014). DNA replicons for plant genome engineering. *Plant Cell* 26 151–163. 10.1105/tpc.113.119792 24443519 PMC3963565

[B4] Baltes N. J. Voytas D. F. (2015). Enabling plant synthetic biology through genome engineering. *Trends Biotechnol.* 33 120–131. 10.1016/j.tibtech.2014.11.008 25496918

[B5] Beetham P. R. Kipp P. B. Sawycky X. L. Arntzen C. J. May G. D. (1999). A tool for functional plant genomics: chimeric RNA/DNA oligonucleotides cause in vivo gene-specific mutations. *Proc. Natl. Acad. Sci. U.S.A.* 96 8774–8778. 10.1073/pnas.96.15.8774 10411951 PMC17592

[B6] Belhaj K. Chaparro-Garcia A. Kamoun S. Patron N. J. Nekrasov V. (2015). Editing plant genomes with CRISPR/Cas9. *Curr. Opin. Biotechnol.* 32 76–84. 10.1016/j.copbio.2014.11.007 25437637

[B7] Bibikova M. Golic M. Golic K. G. Carroll D. (2002). Targeted chromosomal cleavage and mutagenesis in drosophila using zinc-finger nucleases. *Genetics* 161 1169–1175. 12136019 10.1093/genetics/161.3.1169 PMC1462166

[B8] Bogdanove A. J. Voytas D. F. (2011). TAL effectors: customizable proteins for DNA targeting. *Science* 333 1843–1846. 10.1126/science.1204094 21960622

[B9] Budhagatapalli N. Rutten T. Gurushidze M. Kumlehn J. Hensel G. (2015). Targeted modification of gene function exploiting homology-directed repair of TALEN-mediated double-strand breaks in barley. *G*3 5 1857–1863. 10.1534/g3.115.018762 PMC4555222 26153077

[B10] Butler N. M. Baltes N. J. Voytas D. F. Douches D. S. (2016). Geminivirus-mediated genome editing in potato (*Solanum tuberosum* L.) using sequence-specific nucleases. *Front. Plant Sci.* 7:1045 10.3389/fpls.2016.01045 PMC4955380 27493650

[B11] Cai C. Q. Doyon Y. Ainley W. M. Miller J. C. Dekelver R. C. Moehle E. A. (2009). Targeted transgene integration in plant cells using designed zinc finger nucleases. *Plant Mol. Biol.* 69 699–709. 10.1007/s11103-008-9449-7 19112554

[B12] Cermak T. Baltes N. J. Cegan R. Zhang Y. Voytas D. F. (2015). High-frequency, precise modification of the tomato genome. *Genome Biol.* 16: 232 10.1186/s13059-015-0796-9 PMC4635538 26541286

[B13] Chan F. Hauswirth W. W. Wensel T. G. Wilson J. H. (2011). Efficient mutagenesis of the rhodopsin gene in rod photoreceptor neurons in mice. *Nucleic Acids Res.* 39 5955–5966. 10.1093/nar/gkr196 21478169 PMC3152346

[B14] Christian M. Cermak T. Doyle E. L. Schmidt C. Zhang F. Hummel A. (2010). Targeting DNA double-strand breaks with TAL effector nucleases. *Genetics* 186 757–761. 10.1534/genetics.110.120717 20660643 PMC2942870

[B15] Cong L. Ran F. A. Cox D. Lin S. Barretto R. Habib N. (2013). Multiplex genome engineering using CRISPR/Cas systems. *Science* 339 819–823. 10.1126/science.1231143 23287718 PMC3795411

[B16] Dang T. T. Shimatani Z. Kawano Y. Terada R. Shimamoto K. (2013). Gene editing a constitutively active OsRac1 by homologous recombinationbased gene targeting induces immune responses in rice. *Plant Cell Physiol.* 54 2058–2070. 10.1093/pcp/pct147 24158358

[B17] de Pater S. Neuteboom L. W. Pinas J. E. Hooykaas P. J. Van Der Zaal B. J. (2009). ZFN-induced mutagenesis and gene-targeting in *Arabidopsis* through *Agrobacterium*-mediated floral dip transformation. *Plant Biotechnol. J.* 7 821–835. 10.1111/j.1467-7652.2009.00446.x 19754840

[B18] de Pater S. Pinas J. E. Hooykaas P. J. Van Der Zaal B. J. (2013). ZFN-mediated gene targeting of the *Arabidopsis* protoporphyrinogen oxidase gene through *Agrobacterium*-mediated floral dip transformation. *Plant Biotechnol. J.* 11 510–515. 10.1111/pbi.12040 23279135 PMC3719044

[B19] Doudna J. A. Charpentier E. (2014). The new frontier of genome engineering with CRISPR-Cas9. *Science* 346: 1258096 10.1126/science.1258096 25430774

[B20] Endo M. Ishikawa Y. Osakabe K. Nakayama S. Kaya H. Araki T. (2006). Increased frequency of homologous recombination and T-DNA integration in Arabidopsis CAF-1 mutants. *EMBO J.* 25 5579–5590. 10.1038/sj.emboj.7601434 17110925 PMC1679757

[B21] Endo M. Mikami M. Toki S. (2016). Biallelic gene targeting in rice. *Plant Physiol.* 170 667–677. 10.1104/pp.15.01663 26668334 PMC4734577

[B22] Even-Faitelson L. Samach A. Melamed-Bessudo C. Avivi-Ragolsky N. Levy A. A. (2011). Localized egg-cell expression of effector proteins for targeted modification of the *Arabidopsis* genome. *Plant J.* 68 929–937. 10.1111/j.1365-313X.2011.04741.x 21848915

[B23] Fauser F. Roth N. Pacher M. Ilg G. Sanchez-Fernandez R. Biesgen C. (2012). In planta gene targeting. *Proc. Natl. Acad. Sci. U.S.A.* 109 7535–7540. 10.1073/pnas.1202191109 22529367 PMC3358861

[B24] Fauser F. Schiml S. Puchta H. (2014). Both CRISPR/Cas-based nucleases and nickases can be used efficiently for genome engineering in *Arabidopsis thaliana*. *Plant J.* 79 348–359. 10.1111/tpj.12554 24836556

[B25] Feng Z. Mao Y. Xu N. Zhang B. Wei P. Yang D. L. (2014). Multigeneration analysis reveals the inheritance, specificity, and patterns of CRISPR/Cas-induced gene modifications in *Arabidopsis*. *Proc. Natl. Acad. Sci. U.S.A.* 111 4632–4637. 10.1073/pnas.1400822111 24550464 PMC3970504

[B26] Gaj T. Gersbach C. A. Barbas C. F. III (2013). ZFN, TALEN, and CRISPR/Cas-based methods for genome engineering. *Trends Biotechnol.* 31 397–405. 10.1016/j.tibtech.2013.04.004 23664777 PMC3694601

[B27] Gao X. Chen J. Dai X. Zhang D. Zhao Y. (2016). An effective strategy for reliably isolating heritable and Cas9-free *Arabidopsis* mutants generated by CRISPR/Cas9-mediated genome editing. *Plant Physiol.* 171 1794–1800. 10.1104/pp.16.00663 27208253 PMC4936589

[B28] Gao Y. Zhao Y. (2014). Self-processing of ribozyme-flanked RNAs into guide RNAs in vitro and in vivo for CRISPR-mediated genome editing. *J. Integr. Plant Biol.* 56 343–349. 10.1111/jipb.12152 24373158

[B29] Halfter U. Morris P.-C. Willmitzer L. (1992). Gene targeting in *Arabidopsis thaliana*. *Mol. Gen. Genet.* 231 186–193. 1310519 10.1007/BF00279790

[B30] Hartung F. Suer S. Puchta H. (2007). Two closely related RecQ helicases have antagonistic roles in homologous recombination and DNA repair in *Arabidopsis thaliana*. *Proc. Natl. Acad. Sci. U.S.A.* 104 18836–18841. 10.1073/pnas.0705998104 18000056 PMC2141863

[B31] Hiei Y. Ishida Y. Komari T. (2014). Progress of cereal transformation technology mediated by *Agrobacterium tumefaciens*. *Front. Plant Sci.* 5:628 10.3389/fpls.2014.00628 PMC4224067 25426132

[B32] Hrouda M. Paszkowski J. (1994). High fidelity extrachromosomal recombination and gene targeting in plants. *Mol. Gen. Genet.* 243 106–111. 10.1007/BF00283882 8190063

[B33] Jinek M. Chylinski K. Fonfara I. Hauer M. Doudna J. A. Charpentier E. (2012). A programmable dual-RNA–guided DNA endonuclease in adaptive bacterial immunity. *Science* 337 816–821. 10.1126/science.1225829 22745249 PMC6286148

[B34] Kim Y. G. Cha J. Chandrasegaran S. (1996). Hybrid restriction enzymes: zinc finger fusions to Fok I cleavage domain. *Proc. Natl. Acad. Sci. U.S.A.* 93 1156–1160. 10.1073/pnas.93.3.1156 8577732 PMC40048

[B35] Knoll A. Higgins J. D. Seeliger K. Reha S. J. Dangel N. J. Bauknecht M. (2012). The Fanconi anemia ortholog FANCM ensures ordered homologous recombination in both somatic and meiotic cells in *Arabidopsis*. *Plant Cell* 24 1448–1464. 10.1105/tpc.112.096644 22547783 PMC3398556

[B36] Knoll A. Puchta H. (2011). The role of DNA helicases and their interaction partners in genome stability and meiotic recombination in plants. *J. Exp. Bot.* 62 1565–1579. 10.1093/jxb/erq357 21081662

[B37] Kumar S. Alabed D. Worden A. Novak S. Wu H. Ausmus C. (2015). A modular gene targeting system for sequential transgene stacking in plants. *J. Biotechnol.* 207 12–20. 10.1016/j.jbiotec.2015.04.006 25913173

[B38] Kumar S. Barone P. Smith M. (2016). Gene targeting and transgene stacking using intra genomic homologous recombination in plants. *Plant Methods* 12:11 10.1186/s13007-016-0111-0 PMC4736180 26839580

[B39] Kwon Y. I. Abe K. Osakabe K. Endo M. Nishizawa-Yokoi A. Saika H. (2012). Overexpression of OsRecQl4 and/or OsExo1 enhances DSB-induced homologous recombination in rice. *Plant Cell Physiol.* 53 2142–2152. 10.1093/pcp/pcs155 23161853

[B40] Lawrenson T. Shorinola O. Stacey N. Li C. Østergaard L. Patron N. (2015). Induction of targeted, heritable mutations in barley and *Brassica oleracea* using RNA-guided Cas9 nuclease. *Genome Biol.* 16: 258 10.1186/s13059-015-0826-7 PMC4663725 26616834

[B41] Lee K. Y. Lund P. Lowe K. Dunsmuir P. (1990). Homologous recombination in plant cells after *Agrobacterium*-mediated transformation. *Plant Cell* 2 415–425. 10.1105/tpc.2.5.415 2152167 PMC159898

[B42] Li T. Huang S. Jiang W. Z. Wright D. Spalding M. H. Weeks D. P. (2011). TAL nucleases (TALNs): hybrid proteins composed of TAL effectors and FokI DNA-cleavage domain. *Nucleic Acids Res.* 39 359–372. 10.1093/nar/gkq704 20699274 PMC3017587

[B43] Li T. Liu B. Chen C. Y. Yang B. (2016). TALEN-mediated homologous recombination produces site-directed DNA base change and herbicide-resistant rice. *J. Genet. Genomics* 43 297–305. 10.1016/j.jgg.2016.03.005 27180265

[B44] Li Z. Liu Z. B. Xing A. Moon B. P. Koellhoffer J. P. Huang L. (2015). Cas9-guide RNA directed genome editing in soybean. *Plant Physiol.* 169 960–970. 10.1104/pp.15.00783 26294043 PMC4587461

[B45] Lloyd A. Plaisier C. L. Carroll D. Drews G. N. (2005). Targeted mutagenesis using zinc-finger nucleases in *Arabidopsis*. *Proc. Natl. Acad. Sci. U.S.A.* 102 2232–2237. 10.1073/pnas.0409339102 15677315 PMC548540

[B46] Ma X. Zhang Q. Zhu Q. Liu W. Chen Y. Qiu R. (2015). A robust CRISPR/Cas9 system for convenient, high-efficiency multiplex genome editing in monocot and dicot Plants. *Mol. Plant* 8 1274–1284. 10.1016/j.molp.2015.04.007 25917172

[B47] Ma X. Zhu Q. Chen Y. Liu Y. G. (2016). CRISPR/Cas9 platforms for genome editing in plants: developments and applications. *Mol. Plant* 9 961–974. 10.1016/j.molp.2016.04.009 27108381

[B48] Mahfouz M. M. Li L. Shamimuzzaman M. Wibowo A. Fang X. Zhu J. K. (2011). De novo-engineered transcription activator-like effector (TALE) hybrid nuclease with novel DNA binding specificity creates double-strand breaks. *Proc. Natl. Acad. Sci. U.S.A.* 108 2623–2628. 10.1073/pnas.1019533108 21262818 PMC3038751

[B49] Miao Z. H. Lam E. (1995). Targeted disruption of the TGA3 locus in *Arabidopsis thaliana*. *Plant J.* 7 359–365. 10.1046/j.1365-313X.1995.7020359.x 7704051

[B50] Miller J. C. Tan S. Qiao G. Barlow K. A. Wang J. Xia D. F. (2011). A TALE nuclease architecture for efficient genome editing. *Nat. Biotechnol.* 29 143–148. 10.1038/nbt.1755 21179091

[B51] Moritoh S. Eun C. H. Ono A. Asao H. Okano Y. Yamaguchi K. (2012). Targeted disruption of an orthologue of domains rearranged methylase 2, OsDRM2, impairs the growth of rice plants by abnormal DNA methylation. *Plant J.* 71 85–98. 10.1111/j.1365-313X.2012.04974.x 22380881

[B52] Nishizawa-Yokoi A. Nonaka S. Osakabe K. Saika H. Toki S. (2015). A universal positive-negative selection system for gene targeting in plants combining an antibiotic resistance gene and its antisense RNA. *Plant Physiol.* 169 362–370. 10.1104/pp.15.00638 26143254 PMC4577407

[B53] Offringa R. De Groot M. J. Haagsman H. J. Does M. P. Van Den Elzen P. J. Hooykaas P. J. (1990). Extrachromosomal homologous recombination and gene targeting in plant cells after *Agrobacterium* mediated transformation. *EMBO J.* 9 3077–3084. 2209538 10.1002/j.1460-2075.1990.tb07504.x PMC552033

[B54] Okuzaki A. Toriyama K. (2004). Chimeric RNA/DNA oligonucleotide-directed gene targeting in rice. *Plant Cell Rep.* 22 509–512. 10.1007/s00299-003-0698-2 14634786

[B55] Ono A. Yamaguchi K. Fukada-Tanaka S. Terada R. Mitsui T. Iida S. (2012). A null mutation of ROS1a for DNA demethylation in rice is not transmittable to progeny. *Plant J.* 71 564–574. 10.1111/j.1365-313X.2012.05009.x 22448681

[B56] Osakabe K. Nishizawa-Yokoi A. Ohtsuki N. Osakabe Y. Toki S. (2014). A mutated cytosine deaminase gene, codA (D314A), as an efficient negative selection marker for gene targeting in rice. *Plant Cell Physiol.* 55 658–665. 10.1093/pcp/pct183 24371307

[B57] Osakabe Y. Osakabe K. (2015). Genome editing with engineered nucleases in plants. *Plant Cell Physiol.* 56 389–400. 10.1093/pcp/pcu170 25416289

[B58] Osman K. Higgins J. D. Sanchez-Moran E. Armstrong S. J. Franklin F. C. (2011). Pathways to meiotic recombination in *Arabidopsis thaliana*. *New Phytol.* 190 523–544. 10.1111/j.1469-8137.2011.03665.x 21366595

[B59] Ozawa K. Wakasa Y. Ogo Y. Matsuo K. Kawahigashi H. Takaiwa F. (2012). Development of an efficient agrobacterium-mediated gene targeting system for rice and analysis of rice knockouts lacking granule-bound starch synthase (Waxy) and b1,2-xylosyltransferase. *Plant Cell Physiol.* 53 755–761. 10.1093/pcp/pcs016 22327484

[B60] Pacher M. Schmidt-Puchta W. Puchta H. (2007). Two unlinked double-strand breaks can induce reciprocal exchanges in plant genomes via homologous recombination and nonhomologous end joining. *Genetics* 175 21–29. 10.1534/genetics.106.065185 17057227 PMC1775016

[B61] Paszkowski J. Baur M. Bogucki A. Potrykus I. (1988). Gene targeting in plants. *EMBO J.* 7 4021–4026. 16453864 10.1002/j.1460-2075.1988.tb03295.x PMC455109

[B62] Petolino J. F. (2015). Genome editing in plants via designed zinc finger nucleases. *In Vitro Cell. Dev. Biol. Plant* 51 1–8. 10.1007/s11627-015-9663-3 25774080 PMC4352198

[B63] Puchta H. (1998). Repair of genomic double-strand breaks in somatic plant cells by one-sided invasion of homologous sequences. *Plant J.* 13 331–339. 10.1046/j.1365-313X.1998.00035.x

[B64] Puchta H. (2005). The repair of double-strand breaks in plants: mechanisms and consequences for genome evolution. *J. Exp. Bot.* 56 1–14. 15557293 10.1093/jxb/eri025

[B65] Puchta H. Dujon B. Hohn B. (1993). Homologous recombination in plant cells is enhanced by in vivo induction of double strand breaks into DNA by a site-specific endonuclease. *Nucleic Acids Res.* 21 5034–5040. 10.1093/nar/21.22.5034 8255757 PMC310614

[B66] Puchta H. Dujon B. Hohn B. (1996). Two different but related mechanisms are used in plants for the repair of genomic double-strand breaks by homologous recombination. *Proc. Natl. Acad. Sci. U.S.A.* 93 5055–5060. 10.1073/pnas.93.10.5055 8643528 PMC39405

[B67] Puchta H. Fauser F. (2013). Gene targeting in plants: 25 years later. *Int. J. Dev. Biol.* 57 629–637. 10.1387/ijdb.130194hp 24166445

[B68] Puchta H. Fauser F. (2014). Synthetic nucleases for genome engineering in plants: prospects for a bright future. *Plant J.* 78 727–741. 10.1111/tpj.12338 24112784

[B69] Puchta H. Fauser F. (2015). “Double-strand break repair and its application to genome engineering in plants,” in *Advances in New Technology for Targeted Modification of Plant Genomes* eds Zhang F. Puchta H. Thomson J. G. (New York, NY: Springer) 1–20.

[B70] Puchta H. Hohn B. (1991a). The mechanism of extrachromosomal homologous DNA recombination in plant cells. *Mol. Gen. Genet.* 230 1–7. 10.1007/BF00290641 1745222

[B71] Puchta H. Hohn B. (1991b). A transient assay in plant cells reveals a positive correlation between extrachromosomal recombination rates and length of homologous overlap. *Nucleic Acids Res.* 19 2693–2700. 10.1093/nar/19.10.2693 2041745 PMC328188

[B72] Qi Y. Zhang Y. Zhang F. Baller J. A. Cleland S. C. Ryu Y. (2013). Increasing frequencies of site-specific mutagenesis and gene targeting in *Arabidopsis* by manipulating DNA repair pathways. *Genome Res.* 23 547–554. 10.1101/gr.145557.112 23282329 PMC3589543

[B73] Recker J. Knoll A. Puchta H. (2014). The *Arabidopsis thaliana* homolog of the helicase RTEL1 plays multiple roles in preserving genome stability. *Plant Cell* 26 4889–4902. 10.1105/tpc.114.132472 25516598 PMC4311205

[B74] Reiss B. Schubert I. Köpchen K. Wendeler E. Schell J. Puchta H. (2000). RecA stimulates sister chromatid exchange and the fidelity of double-strand break repair, but not gene targeting, in plants transformed by *Agrobacterium*. *Proc. Natl. Acad. Sci. U.S.A.* 97 3358–3363. 10.1073/pnas.97.7.3358 10725370 PMC16244

[B75] Risseeuw E. Offringa R. Franke-Van Dijk M. E. Hooykaas P. J. (1995). Targeted recombination in plants using *Agrobacterium* coincides with additional rearrangements at the target locus. *Plant J.* 7 109–119. 10.1046/j.1365-313X.1995.07010109.x 7894502

[B76] Roth N. Klimesch J. Dukowic-Schulze S. Pacher M. Mannuss A. Puchta H. (2012). The requirement for recombination factors differs considerably between different pathways of homologous double-strand break repair in somatic plant cells. *Plant J.* 72 781–790. 10.1111/j.1365-313X.2012.05119.x 22860689

[B77] Sauer N. J. Narvaez-Vasquez J. Mozoruk J. Miller R. B. Warburg Z. J. Woodward M. J. (2016). Oligonucleotide-mediated genome editing provides precision and function to engineered nucleases and antibiotics in plants. *Plant Physiol.* 170 1917–1928. 10.1104/pp.15.01696 26864017 PMC4825113

[B78] Schiml S. Fauser F. Puchta H. (2014). The CRISPR/Cas system can be used as nuclease for in planta gene targeting and as paired nickases for directed mutagenesis in *Arabidopsis* resulting in heritable progeny. *Plant J.* 80 1139–1150. 10.1111/tpj.12704 25327456

[B79] Schiml S. Puchta H. (2016). Revolutionizing plant biology: multiple ways of genome engineering by CRISPR/Cas. *Plant methods* 12:8 10.1186/s13007-016-0103-0 PMC4730597 26823677

[B80] Shaked H. Melamed-Bessudo C. Levy A. A. (2005). High-frequency gene targeting in Arabidopsis plants expressing the yeast RAD54 gene. *Proc. Natl. Acad. Sci. U.S.A.* 102 12265–12269. 10.1073/pnas.0502601102 16093317 PMC1189313

[B81] Shan Q. Wang Y. Chen K. Liang Z. Li J. Zhang Y. (2013). Rapid and efficient gene modification in rice and *Brachypodium* using TALENs. *Mol. Plant* 6 1365–1368. 10.1093/mp/sss162 23288864 PMC3968307

[B82] Shi J. Gao H. Wang H. Lafitte H. R. Archibald R. L. Yang M. (2016). ARGOS8 variants generated by CRISPR-Cas9 improve maize grain yield under field drought stress conditions. *Plant Biotechnol. J.* 10.1111/pbi.12603 [Epub ahead of print] PMC5258859 27442592

[B83] Shimatani Z. Nishizawa-Yokoi A. Endo M. Toki S. Terada R. (2015). Positive–negative-selection-mediated gene targeting in rice. *Front. Plant Sci.* 5:748 10.3389/fpls.2014.00748 PMC4283509 25601872

[B84] Shrawat A. K. Lorz H. (2006). *Agrobacterium*-mediated transformation of cereals: a promising approach crossing barriers. *Plant Biotechnol. J.* 4 575–603. 10.1111/j.1467-7652.2006.00209.x 17309731

[B85] Shukla V. K. Doyon Y. Miller J. C. Dekelver R. C. Moehle E. A. Worden S. E. (2009). Precise genome modification in the crop species *Zea mays* using zinc-finger nucleases. *Nature* 459 437–441. 10.1038/nature07992 19404259

[B86] Siebert R. Puchta H. (2002). Efficient repair of genomic double-strand breaks by homologous recombination between directly repeated sequences in the plant genome. *Plant Cell* 14 1121–1131. 10.1105/tpc.001727 12034901 PMC150611

[B87] Smith J. Bibikova M. Whitby F. G. Reddy A. R. Chandrasegaran S. Carroll D. (2000). Requirements for double-strand cleavage by chimeric restriction enzymes with zinc finger DNA-recognition domains. *Nucleic Acids Res.* 28 3361–3369. 10.1093/nar/28.17.3361 10954606 PMC110700

[B88] Steinert J. Schiml S. Puchta H. (2016). Homology-based double-strand break-induced genome engineering in plants. *Plant Cell. Rep.* 35 1429–1438. 10.1007/s00299-016-1981-3 27084537

[B89] Sun Y. Zhang X. Wu C. He Y. Ma Y. Hou H. (2016). Engineering herbicide-resistant rice plants through CRISPR/Cas9-mediated homologous recombination of acetolactate synthase. *Mol. Plant* 9 628–631. 10.1016/j.molp.2016.01.001 26768120

[B90] Svitashev S. Young J. K. Schwartz C. Gao H. Falco S. C. Cigan A. M. (2015). Targeted mutagenesis, precise gene editing, and site-specific gene insertion in maize using Cas9 and guide RNA. *Plant Physiol.* 169 931–945. 10.1104/pp.15.00793 26269544 PMC4587463

[B91] Symington L. S. Gautier J. (2011). Double-strand break end resection and repair pathway choice. *Annu. Rev. Genet.* 45 247–271. 10.1146/annurev-genet-110410-132435 21910633

[B92] Terada R. Johzuka-Hisatomi Y. Saitoh M. Asao H. Iida S. (2007). Gene targeting by homologous recombination as a biotechnological tool for rice functional genomics. *Plant Physiol.* 144 846–856. 10.1104/pp.107.095992 17449652 PMC1914187

[B93] Terada R. Urawa H. Inagaki Y. Tsugane K. Iida S. (2002). Efficient gene targeting by homologous recombination in rice. *Nat. Biotechnol.* 20 1030–1034. 10.1038/nbt737 12219079

[B94] Tinland B. Hohn B. Puchta H. (1994). *Agrobacterium tumefaciens* transfers single stranded T-DNA into the plant cell nucleus. *Proc. Natl. Acad. Sci. U.S.A.* 91 8000–8004. 10.1073/pnas.91.17.8000 11607492 PMC44532

[B95] Townsend J. A. Wright D. A. Winfrey R. J. Fu F. Maeder M. L. Joung J. K. (2009). High-frequency modification of plant genes using engineered zinc-finger nucleases. *Nature* 459 442–445. 10.1038/nature07845 19404258 PMC2743854

[B96] Voytas D. F. Gao C. (2014). Precision genome engineering and agriculture: opportunities and regulatory challenges. *PLoS Biol.* 12:e1001877 10.1371/journal.pbio.1001877 PMC4051594 24915127

[B97] Wang Y. Cheng X. Shan Q. Zhang Y. Liu J. Gao C. (2014). Simultaneous editing of three homoeoalleles in hexaploid bread wheat confers heritable resistance to powdery mildew. *Nat. Biotechnol.* 32 947–951. 10.1038/nbt.2969 25038773

[B98] Weeks D. P. Spalding M. H. Yang B. (2016). Use of designer nucleases for targeted gene and genome editing in plants. *Plant Biotechnol. J.* 14 483–495. 10.1111/pbi.12448 26261084 PMC11388832

[B99] Weinthal D. M. Taylor R. A. Tzfira T. (2013). Nonhomologous end joining-mediated gene replacement in plant cells. *Plant Physiol.* 162 390–400. 10.1104/pp.112.212910 23509176 PMC3641218

[B100] Wright D. A. Townsend J. A. Winfrey R. J. Irwin P. A. Rajagopal J. Lonosky P. M. (2005). High-frequency homologous recombination in plants mediated by zinc-finger nucleases. *Plant J.* 44 693–705. 10.1111/j.1365-313X.2005.02551.x 16262717

[B101] Xie K. Minkenberg B. Yang Y. (2015). Boosting CRISPR/Cas9 multiplex editing capability with the endogenous tRNA-processing system. *Proc. Natl. Acad. Sci. U.S.A.* 112 3570–3575. 10.1073/pnas.1420294112 25733849 PMC4371917

[B102] Yamauchi T. Johzuka-Hisatomi Y. Fukada-Tanaka S. Terada R. Nakamura I. Iida S. (2009). Homologous recombination-mediated knock-in targeting of the MET1a gene for a maintenance DNA methyltransferase reproducibly reveals dosage-dependent spatiotemporal gene expression in rice. *Plant J.* 60 386–396. 10.1111/j.1365-313X.2009.03947.x 19519802

[B103] Zetsche B. Gootenberg J. S. Abudayyeh O. O. Slaymaker I. M. Makarova K. S. Essletzbichler P. (2015). Cpf1 is a single RNA-guided endonuclease of a class 2 CRISPR-Cas system. *Cell* 163 759–771. 10.1016/j.cell.2015.09.038 26422227 PMC4638220

[B104] Zhang F. Maeder M. L. Unger-Wallace E. Hoshaw J. P. Reyon D. Christian M. (2010). High frequency targeted mutagenesis in *Arabidopsis thaliana* using zinc finger nucleases. *Proc. Natl. Acad. Sci. U.S.A.* 107 12028–12033. 10.1073/pnas.0914991107 20508152 PMC2900673

[B105] Zhang F. Voytas D. F. (2011). Targeted mutagenesis in Arabidopsis using zinc-finger nucleases. *Methods Mol. Biol.* 701 167–177. 10.1007/978-1-61737-957-4_9 21181530

[B106] Zhang Y. Zhang F. Li X. Baller J. A. Qi Y. Starker C. G. (2013). Transcription activator-like effector nucleases enable efficient plant genome engineering. *Plant Physiol.* 161 20–27. 10.1104/pp.112.205179 23124327 PMC3532252

[B107] Zhou H. Liu B. Weeks D. P. Spalding M. H. Yang B. (2014). Large chromosomal deletions and heritable small genetic changes induced by CRISPR/Cas9 in rice. *Nucleic Acids Res.* 42 10903–10914. 10.1093/nar/gku806 25200087 PMC4176183

[B108] Zhu T. Mettenburg K. Peterson D. J. Tagliani L. Baszczynski C. L. (2000). Engineering herbicide-resistant maize using chimeric RNA/DNA oligonucleotides. *Nat. Biotechnol.* 18 555–558. 10.1038/75435 10802626

